# Genetic and neuronal basis for facial emotion perception in humans and macaques

**DOI:** 10.1093/nsr/nwae381

**Published:** 2024-11-08

**Authors:** Li Wang, Bo Zhang, Xiqian Lu, Ruidi Wang, Jian Ma, Yujie Chen, Yuan Zhou, Ji Dai, Yi Jiang

**Affiliations:** State Key Laboratory of Brain and Cognitive Science, Institute of Psychology, Chinese Academy of Sciences, Beijing 100101, China; Department of Psychology, University of Chinese Academy of Sciences, Beijing 100049, China; Shenzhen Technological Research Center for Primate Translational Medicine, Shenzhen-Hong Kong Institute of Brain Science, Shenzhen Institute of Advanced Technology, Chinese Academy of Sciences, Shenzhen 518055, China; Key Laboratory of Brain Science, Zunyi Medical University, Zunyi 563000, China; State Key Laboratory of Brain and Cognitive Science, Institute of Psychology, Chinese Academy of Sciences, Beijing 100101, China; Department of Psychology, University of Chinese Academy of Sciences, Beijing 100049, China; State Key Laboratory of Brain and Cognitive Science, Institute of Psychology, Chinese Academy of Sciences, Beijing 100101, China; Department of Psychology, University of Chinese Academy of Sciences, Beijing 100049, China; Shenzhen Technological Research Center for Primate Translational Medicine, Shenzhen-Hong Kong Institute of Brain Science, Shenzhen Institute of Advanced Technology, Chinese Academy of Sciences, Shenzhen 518055, China; CAS Key Laboratory of Brain Connectome and Manipulation, Brain Cognition and Brain Disease Institute, Shenzhen Institute of Advanced Technology, Chinese Academy of Sciences, Shenzhen 518055, China; State Key Laboratory of Brain and Cognitive Science, Institute of Psychology, Chinese Academy of Sciences, Beijing 100101, China; Department of Psychology, University of Chinese Academy of Sciences, Beijing 100049, China; State Key Laboratory of Brain and Cognitive Science, Institute of Psychology, Chinese Academy of Sciences, Beijing 100101, China; Department of Psychology, University of Chinese Academy of Sciences, Beijing 100049, China; Shenzhen Technological Research Center for Primate Translational Medicine, Shenzhen-Hong Kong Institute of Brain Science, Shenzhen Institute of Advanced Technology, Chinese Academy of Sciences, Shenzhen 518055, China; CAS Key Laboratory of Brain Connectome and Manipulation, Brain Cognition and Brain Disease Institute, Shenzhen Institute of Advanced Technology, Chinese Academy of Sciences, Shenzhen 518055, China; Guangdong Provincial Key Laboratory of Brain Connectome and Behavior, Shenzhen Institute of Advanced Technology, Chinese Academy of Sciences, Shenzhen 518055, China; Department of Psychology, University of Chinese Academy of Sciences, Beijing 100049, China; State Key Laboratory of Brain and Cognitive Science, Institute of Psychology, Chinese Academy of Sciences, Beijing 100101, China; Department of Psychology, University of Chinese Academy of Sciences, Beijing 100049, China

**Keywords:** facial emotion recognition, behavioral genetics, twin study, spatial frequency, amygdala

## Abstract

The ability to rapidly recognize basic facial emotions (e.g. fear) is crucial for social interactions and adaptive functioning. To date, the origin of facial-emotion-recognition ability remains equivocal. Using a classical twin design in humans, we found a clear dissection of low and high spatial frequencies (LSF and HSF) in facial emotion perception: whereas genetic factors contributed to individual variation in LSF processing, HSF processing is largely shaped by environmental effects. Furthermore, the ability to recognize facial emotions of LSF content genetically correlated with the function of the amygdala. Crucially, single-unit recording of the amygdala in macaques further revealed the dissociation between LSF and HSF processing in facial emotion perception, indicating the existence of an evolutionarily conserved mechanism. This cross-species study enhances insights into the neurobiological dual-route model (subcortical vs. cortical) of emotion perception and illuminates the origin and the functional development of the emotional brain in primates.

## INTRODUCTION

In daily social exchanges, emotions are conveyed through gestures, the voice, and most importantly, facial expressions. The ability to rapidly recognize basic facial emotions (e.g. happiness or fear) plays a foundational role in interpersonal interactions and adaptive functioning, as it assists humans in evaluating the other person's affective state and thus prepares them for appropriate subsequent behaviors (e.g. approach or avoid) [1,2]. This fundamental human capability emerges very early in life [3] and is essentially universal across many different cultures [4]. However, individuals exhibit significant variation in their ability to recognize facial emotions. Impairments in this ability have been implicated in genetic social cognitive disorders characterized by striking social deficits, such as autism and schizophrenia [5–7]. Given its strong association with social deficits, understanding the origin of individual variability in facial-emotion-recognition ability is central to research on the etiology of social cognitive disorders. It is worth noting that facial emotion recognition is supported by the amygdala, an evolutionarily conserved emotional structure that is genetically regulated and largely shared with non-human species [8–11]. Indeed, the ability to extract facial expression information is also present in monkeys [12,13], suggesting an evolutionary basis for the mechanism of facial emotion recognition. Based on the aforementioned evidence, it is reasonable to hypothesize that such an evolutionarily adaptive ability may be strongly shaped by genes and hard-wired in the primate brain. However, currently, it remains equivocal whether facial-emotion-recognition ability is genetically determined or merely nurtured through extensive postnatal social experience.

The present study thus aimed to bridge this gap by investigating the heritability of facial-emotion-recognition ability using a behavioral genetic methodology [14]. In particular, we manipulated one basic visual attribute of the emotional faces (spatial frequency information) and specifically examined the respective roles of the two essential spatial frequency components, namely low- (LSF) and high-spatial-frequency (HSF) information [15,16], in facial emotion processing. A wealth of research has demonstrated that spatial frequencies play a crucial role in various aspects of perception, including object recognition, face processing and particularly emotion perception [17–22]. It has been documented that the LSF component contains global configurational properties that are preferentially transmitted to the subcortical regions (e.g. the amygdala) by the magnocellular pathway at a rapid speed, which is sufficient to enable fast and coarse perception of emotional expressions [15,23]. In contrast, the HSF component conveys localized fine-grained features and is transferred via the parvocellular pathway at a relatively slow speed to the ventral visual cortex, which is important for precise recognition of gender and identity. Although the LSF component has long been assumed to play a special role in the early and successful extraction of facial affect [15,24,25], this view is being challenged by divergent findings reflecting that the use of spatial frequency information in emotion perception is flexible and the HSF component is also involved in the processing of facial expressions [9,26–28]. Here, we sought to pinpoint the differential contributions of LSF and HSF components to facial emotion processing from a genetic perspective.

**Figure 1.**
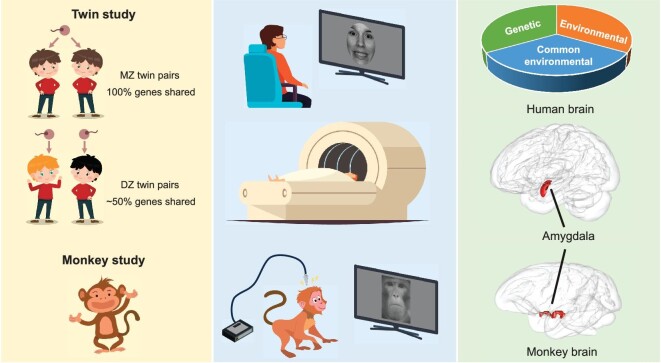
Schematic illustration of the twins-based behavioral experiment, the twins-based fMRI experiment and the monkey electrophysiological experiment. Twin participants performed the emotion and gender discrimination tasks in the behavioral experiment. A subset of these twins also took part in the fMRI experiment, in which the structure and function of the amygdala were measured. Additionally, single-unit responses to different facial expressions from the amygdala of two awake monkeys were recorded.

To this end, we decomposed facial expressions into LSF and HSF components and investigated the emotional recognition of different spatial frequency contents in a group of twin participants (Fig. 1). By tailoring stimuli that primarily engage just one of the two pathways (subcortical-magnocellular and cortical-parvocellular), we were able to address the specific functional properties of the partly segregated pathway to the amygdala and reveal in more detail the origin of facial-emotion-recognition ability. Motivated by the fact that the amygdala is an inherited central node for emotion [8,29,30], we further explored the genetic overlap of facial-emotion-recognition ability with the structure and the function of the amygdala (Fig. 1).

Moreover, we further examined whether the dissociation between LSF and HSF components is phylogenetically ancient and can be observed in non-human primates (e.g. monkeys). Due to their close genetic relationship with humans, non-human primates have been used for *in-vivo* electrophysiological recording for decades, providing extra details at the cellular scale for a specific brain region. The aim here was to investigate whether the amygdala neurons in macaque monkeys are also sensitive to spatial frequency components of facial emotion and further establish the links between different spatial frequency components (LSF and HSF) and the neuronal responses of the amygdala in facial emotion perception (Fig. 1). More importantly, if the genetic and neuronal dissections of LSF and HSF information processing are similarly observed in both human and macaque amygdala, it will provide compelling evidence for the existence of an evolutionarily conserved spatial-frequency-dependent mechanism for facial emotion recognition in the primate brain.

## RESULTS

### Genetic dissection of LSF and HSF components in facial emotion perception

**Figure 2.**
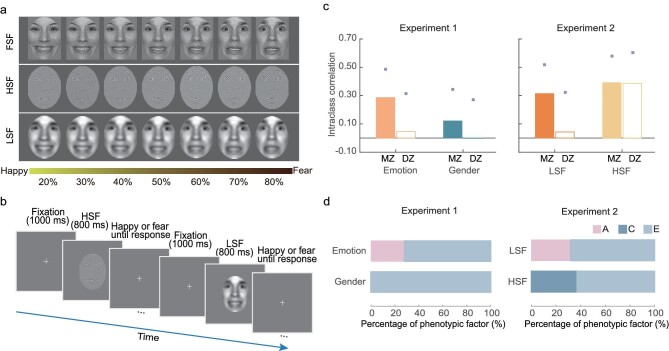
Experimental design and results for the twins-based behavioral experiment. (a) Sample stimuli of the morph continua used in Experiment 1 (full bandwidth, FSF) and Experiment 2 (HSF and LSF). The abscissa represents the degree of expressivity of fear. (b) Schematic representation of the experimental procedure. (c) Intraclass correlations for MZ and DZ twins. In Experiment 1, for emotion discrimination ability, the intraclass correlations of MZ exceeded the DZ correlations. However, MZ twin pairs did not show a significant difference from DZ twin pairs in facial-gender-discrimination ability. In Experiment 2, MZ twin pairs exhibited greater similarity in emotion discrimination ability for LSF faces compared to DZ twin pairs, whereas the emotion discrimination ability of HSF faces showed no difference in intraclass correlation between MZ and DZ twin pairs. The small squares indicate upper boundaries of 95% CIs for intraclass correlations. (d) Proportions of the observed phenotypic variance attributable to additive genetic, common environmental and unique environmental factors. In Experiment 1, univariate genetic analyses revealed the heritability of emotion discrimination ability, but no evidence of heritability was observed with gender discrimination ability. In Experiment 2, there were reliable genetic influences on the emotion discrimination ability for LSF faces. By contrast, the emotion discrimination ability for HSF faces was mainly accounted for by environmental effects.

Twin participants were recruited in the behavioral experiments (see Materials and Methods for more detail regarding the participant information). In Experiment 1, facial emotion and facial-gender-discrimination abilities were tested in a sample of monozygotic (MZ) and dizygotic (DZ) twin pairs (Fig. 2a and b). The assumption underlying the twin design is that both MZ and DZ twins share their environment to the same degree, while MZ twins share 100% and DZ twins share only 50% of their genetic material on average. Therefore, if genetic factors play a role in facial emotion perception, MZ twin pairs’ emotion discrimination ability should be more similar than that of DZ twin pairs. Indeed, intraclass correlation analyses showed that the facial-emotion-discrimination ability (measured by the difference limen) was more similar in MZ twins than in DZ twins (0.286 vs. 0.045, Fig. 2c), indicating substantial genetic influences on facial-emotion-discrimination ability. Further univariate genetic analyses revealed that the heritability of the facial-emotion-discrimination ability was 26% (95% confidence interval (CI), 3%–47%, Fig. 2d). By contrast, MZ twin pairs showed a relatively small difference from DZ twin pairs in facial-gender-discrimination ability (0.121 vs. -0.002, Fig. 2c), and univariate genetic analyses revealed no heritability of this ability (Fig. 2d).

Experiment 1 demonstrates that facial emotion but not facial-gender-discrimination ability is heritable. However, the role of spatial frequency content in the observed genetic influences on facial-emotion-discrimination ability remains unknown. In Experiment 2, LSF and HSF faces were employed to further explore the differential contributions of LSF and HSF contents. For the emotion discrimination ability of LSF faces, the intraclass correlation of MZ twin pairs exceeded the DZ correlation (0.314 vs. 0.043, Fig. 2c) and the heritability was estimated to be 30% (CI, 5%–51%, Fig. 2d), consistent with the findings observed in Experiment 1. In contrast to the emotion discrimination ability of LSF faces, there was no difference between the intraclass correlations of MZ and DZ twins when HSF faces were used as test stimuli (0.391 vs. 0.387, Fig. 2c), indicating the absence of genetic influences on the emotion discrimination ability of HSF faces. Instead, common environmental factors could explain 38% (CI, 21%–53%, Fig. 2d) of the phenotypic variation, suggesting that environmental factors play a vital role in shaping the emotion discrimination ability of HSF faces. These findings together demonstrate that genetic factors specifically influence the emotion discrimination ability of LSF but not HSF faces.

### Functional dissection of LSF and HSF facial emotion perception in the human amygdala

**Figure 3.**
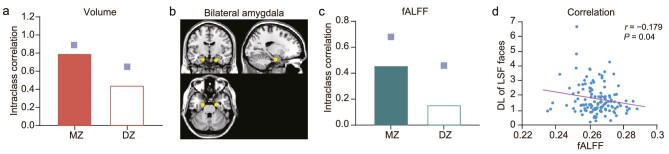
The intraclass correlation for MZ and DZ in the amygdala. (a) Intraclass correlations for the amygdala's volume in MZ and DZ twins. (b) Bilateral amygdala seed ROIs. The left side corresponds to the left hemisphere. (c) Intraclass correlations for the amygdala's fALFF in MZ and DZ twins. (d) The correlation between fALFF and emotion discrimination ability for LSF faces. The small squares indicate upper boundaries of 95% CIs for intraclass correlations.

Given that the amygdala is an inherited central node for emotion perception [8,29,30], we specifically explored the genetic relationship between the amygdala and facial-emotion-discrimination ability from structural MRI images and resting-state functional MRI images. A portion of the twin participants from the behavioral experiment also took part in the fMRI experiment. Among these participants, the heritability for LSF processing in facial emotion perception is 30%, and that for HSF processing is 0%. Intraclass correlation analyses showed that MZ twins exhibited greater similarity for amygdala volume compared to DZ twins (0.794 vs. 0.443, Fig. 3a), indicating substantial genetic influences on amygdala volume. Univariate genetic analyses revealed that the heritability of amygdala volume was 77% (95% CI, 63%–86%). However, the amygdala volume neither correlated with the emotion discrimination ability of LSF faces (*r* = −0.029, *P* = 0.76) nor with that of HSF faces (*r* = 0.129, *P* = 0.13).

We further examined the link between the function of the amygdala and facial-emotion-discrimination ability. Previous research has shown that the fractional amplitude of low-frequency fluctuation (fALFF) in the amygdala is enhanced in patients with emotional perception disorders, indicating that fALFF is negatively associated with emotional perception abilities [31,32]. Intriguingly, we found a genetic influence on the fALFF of the amygdala (Fig. 3c). The intraclass correlations revealed that the fALFF of the amygdala was more similar in MZ twins than in DZ twins (0.457 vs. 0.155, Fig. 3c). Further univariate genetic analyses revealed that the heritability of the amygdala's fALFF was 42% (95% CI, 16%–62%, Table S1). More importantly, the correlation between the fALFF of the amygdala and the emotion discrimination ability of LSF faces was significant (*r* = −0.179, *P* = 0.04; Fig. 3d). On the contrary, the emotion discrimination ability of HSF faces had no significant correlation with the fALFF of the amygdala (*r* = −0.036, *P* = 0.68). Further bivariate genetic analyses found that 100% of the phenotypic association between the fALFF of the amygdala and the emotion discrimination ability of LSF faces was accounted for by shared genetic influences, highlighting the existence of genetic pleiotropy between these two phenotypes.

### Neuronal dissection of LSF and HSF components in the monkey amygdala

**Figure 4.**
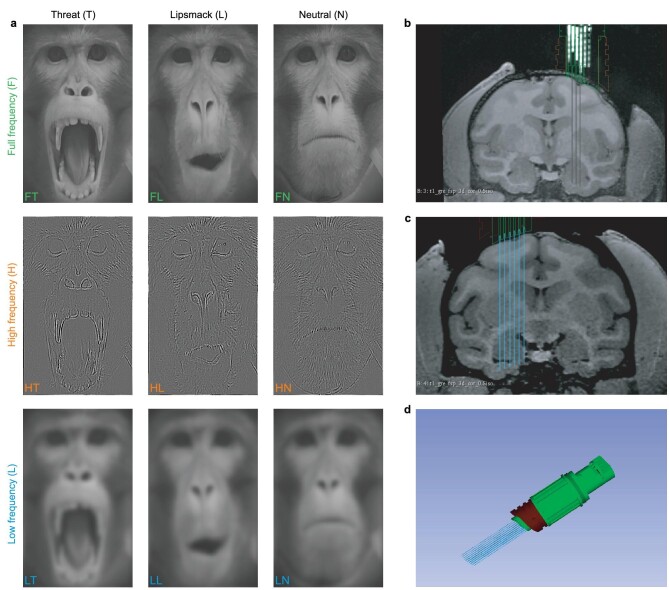
Illustration of visual stimuli and amygdala recording. (a) Faces of different expressions and spatial frequency. Rows from top to bottom: full/high/low spatial frequency; columns from left to right: threat/lipsmack/neutral expression. Each condition is represented by a combination of the first letter in the row and column (e.g. FT represents Full frequency and Threat expression). (b and c) The location of the implanted micro-drive and the representative recording electrodes. (d) A schematic of the micro-drive used in the current experiment.

To further clarify the contributions of LSF and HSF components to facial expression processing in the amygdala at the neuronal level, we recorded single-unit responses to different facial expressions [12] with different spatial frequencies (LSF, HSF and Broadband) from the amygdala of two awake monkeys (Fig. 4a). A semi-chronic micro-drive with 32 recording channels was implanted above the amygdala to allow long-term and easy access to the whole amygdala (Fig. 4b and c) [33]. An illustration of the micro-drive is shown in Fig. 4d. The monkey facial expressions included an affiliative expression (lipsmack), a neutral expression and an aggressive expression (open-mouth threat) (Fig. 4a). A lipsmack is an appeasing expression used by monkeys during a friendly approach, preceding grooming, or as part of a complex of gestures used to beg for food or grooming from other monkeys or caretakers [34]. The open-mouth threat is a typical aggressive facial expression [34].

**Figure 5.**
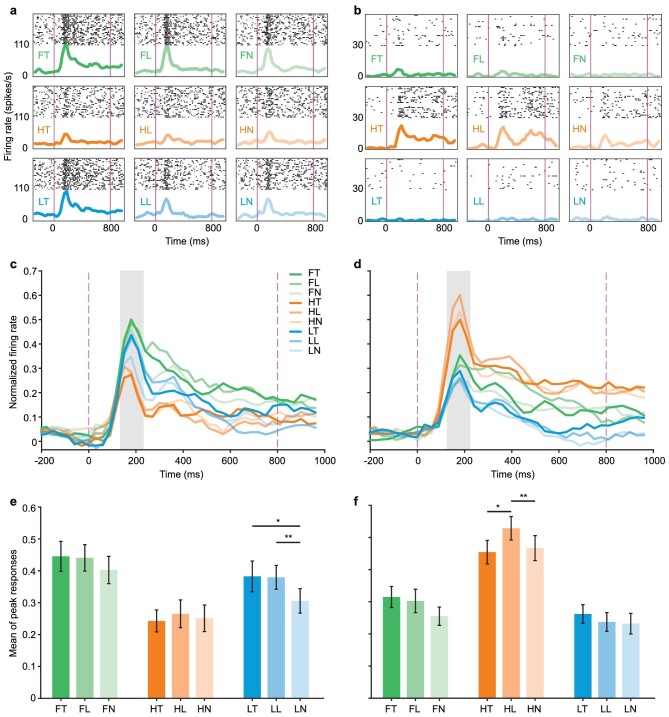
Neural responses to facial expressions. (a) Example of an LSF-preferred neuron that showed stronger responses to LSF facial expressions than to HSF faces. (b) Example of an HSF-preferred neuron that showed stronger responses to HSF facial expressions than to LSF faces. The condition in each row and column is consistent with that in Fig. 1 and labeled with the same ID. The black dots are the raster plots of the spikes; the thick curves are the spike density functions; the vertical magenta lines are the onset and offset of visual stimuli. (c) The mean firing rate after normalization for the LSF-preferred group. (d) The mean firing rate after normalization for the HSF-preferred group. (e) Mean responses during the peak (indicated by the shadow in (c)) for the LSF-preferred group. (f) Mean responses during the peak (indicated by the shadow in (d)) for the HSF-preferred group. Error bars indicate standard error of mean. *: *P* < 0.05; **: *P* < 0.01 (paired *t-*test).

We recorded 82 amygdala cells that responded to at least one type of emotional face and found that each cell showed differential response properties to different facial expressions and/or spatial frequencies (SFs). Specifically, a portion of the cells showed stronger responses to LSF facial expressions than to HSF faces (Fig. 5a), while the others responded oppositely (Fig. 5b), pointing to two distinct neuron populations. We then classified these cells into the LSF-preferred group and HSF-preferred group according to their preferred responses to LSF and HSF facial expressions. A typical cell in the LSF-preferred group normally showed strong responses to both full and LSF facial expressions but weak responses to HSF facial expressions (Fig. 5a). In contrast, many cells in the HSF-preferred group normally only responded strongly to HSF facial expressions but showed weak responses even to full SF facial expressions (Fig. 5b). More intriguingly, within the LSF or HSF faces, the response variation across different facial expressions seemed to be subtle.

To further elucidate the influence of SFs on emotion processing, we next calculated the population responses for the LSF-preferred and the HSF-preferred groups. From the mean firing rates for the LSF-preferred cells (*n* = 36), we noticed the peak responses to the LSF neutral faces were smaller than those to the LSF threat and LSF lipsmack faces (Fig. 5c), so we extracted the responses from the peri-peak period (130 to 230 ms after stimulus presentation) and calculated the average responses for all conditions. As shown in Fig. 5e, the mean peak response to LSF neutral faces (0.31 ± 0.04, mean ± SE) was significantly smaller than that to LSF threat (0.38 ± 0.05, *t*(35) = 2.37, *P* = 0.02) and LSF lipsmack faces (0.38 ± 0.04, *t*(35) = −3.27, *P* = 0.002). The overall responses were commensurate for full and LSF but not for HSF faces. In contrast, cells in the HSF-preferred (*n* = 46) group showed strong responses only to the HSF faces (yellow lines in Fig. 5d). The mean peak responses (Fig. 5f) indicated that such cells preferred positive expression (HL = 0.53 ± 0.04) relative to negative expression (HT = 0.45 ± 0.04, *t* (45) = −2.37, *P* = 0.02) and neutral expression (HN = 0.47 ± 0.04, *t*(45) = −3.15, *P* = 0.003). Taken together, these data suggested that LSF- and HSF-preferred cells might play different roles in encoding facial emotion. In particular, the LSF-preferred cells conveyed both threat and positive emotions while the HSF-preferred cells conveyed only positive emotion, suggesting that the threat signal is mainly encoded by the LSF-preferred cells in the amygdala.

Finally, we investigated whether the LSF and HSF emotions were encoded by distinct subregions or by overlapping regions in the amygdala. We reconstructed the recording sites from the recording coordinates. We found that the LSF- and HSF-preferred cells were interleaved with each other (Fig. S2). Therefore, these results indicate that the amygdala may not contain a special subregion that separately and specifically processes either LSF or HSF emotional inputs. Taken together, the current findings obtained from both human behavioral genetic and neuroimaging research as well as from monkey neurophysiological experiments suggest that during the brain developmental process, the LSF- and HSF-preferred processing, though respectively shaped by genetic and environmental factors, may work in tandem to shape the functional development and maturation of the emotional brain in primates.

## DISCUSSION

Humans possess a remarkable ability to rapidly recognize facial emotions, allowing the exchange of information regarding internal states and facilitating appropriate social interaction. Given its highly adaptive significance, it is not surprising that this facial-emotion-recognition ability is evident even in young infants and non-human primates (e.g. macaque monkeys) [3,12,13], indicating that it may be hardwired in the primate brain. The current study assessed the facial-emotion-recognition ability in twin participants and found robust heritability of such an ability. By contrast, no evidence of heritability is obtained for the facial gender recognition ability. This distinction between the origins of facial-emotion and facial-gender-recognition abilities indicates independent processing routes for functionally different types of facial information (e.g. emotion and gender) [35]. By further manipulating the spatial frequency content of facial expressions via high-pass and low-pass filtering, we found that the ability to recognize facial emotions of LSF content is highly influenced by genetic disposition whereas the ability to recognize facial emotions of HSF content is largely shaped by environmental factors. More critically, the emotional recognition of LSF but not HSF content shares a common genetic origin with the function, but not the structure, of the amygdala. These findings together provide direct empirical evidence for the dissociation between the LSF and HSF components from a genetic perspective, thereby offering a differentiated solution to the ‘nature-or-nurture’ problem of facial-emotion-recognition ability [36]. Additionally, such dissociation is conserved across species and can also be observed in non-human primates, with a population of monkey amygdala neurons being sensitive to the LSF component and another population to the HSF component.

Previous studies examining the differential contributions of LSF and HSF contents to facial emotion processing have yielded mixed results. Some evidence revealed an LSF advantage in the perception of facial affect [24,25]. Conversely, reports from other studies have demonstrated that observers might be more biased in favor of HSF than LSF when asked to detect facial expressions [28]. The neuroimaging findings are also inconsistent, as some studies reported that the LSF component of a fearful facial expression produced more activation in the amygdala compared to the HSF component but some others found similar amygdala responses to LSF and HSF components [15,37,38]. There is still a lack of empirical evidence that could well reconcile these controversial findings. Using behavioral genetic methods, the current study demonstrates a reliable genetic contribution to individual variation in facial emotion recognition of LSF content but not HSF content, with the latter substantially explained by environmental effects, providing clear evidence for the distinction between LSF and HSF processing. This resonates well with developmental studies showing that face recognition in newborns is solely based on LSF but not HSF information [39]. Despite the involvement of both LSF and HSF contents in emotion processing, the genetic roots and underlying processes may be quite different for these two spatial frequency components [40]. The ability to recognize facial emotions of HSF content may be obtained through ontogenesis and occur as a result of long-term learning. In a different way, the ability to recognize facial emotions of LSF content may mainly be acquired phylogenetically and mediated by an innate and genetically determined module.

Notably, the dissociation of the heritability of LSF and HSF processing contributes to the understanding of the neurobiological dual-route model of the emotional brain [41]. This model argues for two parallel routes for emotional information processing: a short quick route from the sensory thalamus directly to the amygdala performing fast and coarse processing, and a slower and more detailed processing route, indirectly via cortical areas, to the amygdala. While the former subcortical pathway draws primarily on magnocellular input and is selectively tuned to the LSF component, the latter cortical pathway receives substantial parvocellular input and responds chiefly to the HSF component. By exploiting the distinct sensitivities of these two pathways to different ranges of SFs, our study provides new insights into the origin and the functional development of the human emotional brain. The heritability of LSF processing and its genetic link with the function of the amygdala offer evidence that the subcortical pathway primarily dedicated to evolutionary prepared affective stimuli might be inherent and phylogenetically ancient. Conversely, the learned mechanism for HSF processing implies that the cortical pathway may be ontogenetically acquired. Together, our findings provide clear evidence that genetic and environmental factors may work in tandem to shape the functional development and maturation of the emotional brain. It is conceivable that the subcortical pathway serves to rapidly direct one's attention towards facial expressions (e.g. fear or happiness) from birth, which aids in the fast detection of potential danger or reward in the environment. The cortical pathway may then be developed via learning and is responsible for the detailed analyses of emotional information, providing adaptive advantages in complex emotional situations.

More critically, the dissociation between LSF and HSF processing in the amygdala is not only observed in humans but also in non-human primates, providing compelling evidence for the existence of an evolutionarily conserved mechanism for facial emotion recognition. By using electrophysiological recordings in monkeys, we found that a particular cluster of amygdala neurons displayed sensitivity to LSF facial expression, while a separate cluster responded to HSF facial expression. Since previous human fMRI results can only reflect responses on a coarse scale, the monkey neurophysiological data obtained here can provide further details at the cellular level, pointing out the cell population specifically encoding LSF facial expression. In addition, LSF-preferred cells show greater responses to both positive and negative expressions compared to neutral expressions for LSF faces; whereas HSF-preferred cells only show a preference for positive expression. This finding is consistent with a former human fMRI study showing that amygdala responses to fearful expressions are greater for intact or LSF faces than for HSF faces [15]. According to the dual-route hypothesis, fast and coarse information is mainly processed via the subcortical pathway. Since negative signals possess higher priority in survival, the observation that negative expression is mainly encoded by the LSF-preferred cells in the amygdala implies that these cells are likely mediated by the magnocellular pathway. However, we discovered that LSF and HSF processing do not engage distinct subregions in the amygdala. Further neuronal tracing studies are still needed to clarify LSF- and HSF-cell origins. It is conceivable that during the brain developmental process, LSF-preferred and HSF-preferred processing, though respectively shaped by genetic and environmental factors, may work together to shape the functional development and maturation of the emotional brain.

Finally, several limitations of the current study should be acknowledged. First, incorporating emotional hybrid faces [42–47] in future twin studies may facilitate a fair comparison between LSF and HSF processing, potentially delineating whether a dissociation exists between these two components in terms of unconscious emotion recognition. Second, the issue of hemispheric asymmetry in relation to positive versus negative emotional valence is crucial in the field of emotion recognition [48–51], yet it remains an unresolved question. It is important to pursue additional investigations that will gather more data and provide thorough analyses to fill this gap. Additionally, future twin studies with larger sample sizes are necessary to corroborate the current findings. Larger sample sizes will help to reduce the risk of false positives and increase the generalizability of our results.

### CONCLUSION

In conclusion, the current study clearly demonstrates that facial emotion recognition is heritable and hardwired in the human brain. Notably, while genetic factors play a significant role in explaining individual variation in the emotional recognition of LSF, the processing of HSF is predominantly influenced by environmental factors. Furthermore, the emotional recognition of LSF is genetically linked to the function of the amygdala, whereas this association is not observed for HSF processing. Additionally, the demarcation between LSF and HSF processing is conserved across species and can also be observed in non-human primates. Collectively, these findings offer empirical support for the across-species dissociation between LSF and HSF processing in terms of genetics and neuronal populations, providing fresh insights into the origin and the functional development of the emotional brain in primates.

## MATERIALS AND METHODS

### Twins-based behavioral experiment

#### Participants

Seventy MZ and 51 DZ twin pairs (a total of 242 participants) participated in behavioral Experiment 1, and 64 MZ and 47 DZ twin pairs (a total of 222 participants) took part in behavioral Experiment 2. All twin pairs were from BeTwiSt, which is a longitudinal study of twins in Beijing, China [52]. We recruited as many twins as possible. They had normal or corrected-to-normal visions and were naive to the purpose of the experiments. They all gave written informed consent following procedures and protocols approved by the institutional review board of the Institute of Psychology, Chinese Academy of Sciences.

#### Stimuli

Stimuli were generated and displayed using MATLAB (Mathworks, Inc.) together with Psychophysics Toolbox extensions. Two faces (one female and one male) displaying happy and fearful expressions were taken from the NimStim face-stimulus set [53]. They were then converted to grayscale and cropped to an oval shape to remove features outside of the face (e.g. hair and ears). In Experiment 1, two sets of morphed faces were employed: one morphed from a fearful female face to a happy male face, and the other set from a happy female face to a fearful male face. The fearful female face was morphed along a continuum with the happy male face using Abrosoft FantaMorph software (https://www.fantamorph.com/). The 0% and 100% morph levels corresponded to the prototypical happy male face or fearful female face respectively, and the 50% morph level corresponded to a morphed image with equal weighting of both faces. Seven levels were used: 20, 30, 40, 50, 60, 70 and 80%. The happy female face and the fearful male face were morphed in the same manner. In Experiment 2, two continua of morphed faces were created by blending the happy and fearful expressions of the same identity (female or male) in 10% increments ranging from 20% to 80% expressivity of fear to obtain seven levels. Next, these two sets of morphed faces were filtered to create LSF and HSF faces. Spatial frequency content was manipulated using a second-order Butterworth filter with a low-pass cut-off of fewer than two cycles per degree for the LSF faces and a high-pass cut-off of more than six cycles per degree for HSF faces, following previous studies [28,54].

#### Procedure for behavioral experiment

In behavioral Experiment 1, stimuli were displayed on a 19-inch Cathode Ray Tube monitor, and the viewing distance was 80 cm. Each trial began with a fixation on a central cross (0.5° × 0.5°) within a white frame (12.6° × 12.6°). After 1 s, a morphed face (subtended approximately 3.2° × 4.2° in visual angle) appeared in the center of the screen for 800 ms. Then observers were required to make a four-alternative forced choice to indicate, as accurately as possible, which type of face was shown (female fearful, male fearful, female happy or male happy). Each observer completed 140 trials with 20 trials in each of the seven test conditions (20, 30, 40, 50, 60, 70 and 80% expressivity of fear).

Behavioral Experiment 2 followed the same design and procedure as Experiment 1, except that LSF and HSF faces were used as test stimuli and observers had to make a two-alternative forced choice (fearful or happy). Experiment 2 consisted of 280 trials, with 140 trials for the LSF condition and 140 trials for the HSF condition.

### Twins-based MRI experiment

Thirty-eight MZ and 35 DZ twin pairs (20.4 ± 2.3 years old, mean ± SD; 50.7% were women) involved in behavioral Experiment 2 also participated in the MRI experiment, in which structural images and resting-state functional images were collected. Structural and functional MRI scans were collected using a General Electric 3.0T MRI scanner at the Magnetic Resonance Imaging Research Center, Institute of Psychology, Chinese Academy of Sciences. The regions of interest (ROIs) of the amygdala (Fig. 3b) were identified as spheres with a radius of 6 mm centered on Montreal Neurological Institute coordinates of (20, −10, −30) and (−20, −10, −28) [15]. The low-frequency fluctuations were performed using DPARSF software (see supplementary information for more details).

### Monkey electrophysiological experiment

#### Animals

Two adult male macaque monkeys were used in the experiment. All monkeys were housed in a primate facility accredited by the Association for Assessment and Accreditation of Laboratory Animal Care (AAALAC) with environmental control. All experimental procedures were approved by the Institutional Animal Care and Use Committee (IACUC) at Shenzhen Institutes of Advanced Technology, Chinese Academy of Sciences, following the guidelines stated in the Guide for Care and Use of Laboratory Animals (Eighth Edition, 2011).

#### Visual stimulus and electrophysiological recording

Emotional monkey faces including three expressions (Threat/Lipsmack/Neutral) and three spatial frequencies (Full/High/Low-SF) were used in the experiment [12,16,27]. For the LSF and HSF faces, the SF was filtered at lower than 0.8 cycles/degree or higher than four cycles/degree. All stimuli were balanced in brightness and contrast. During the experiment, the monkey was trained to sit quietly in a chair with the head fixed. Visual stimuli were presented on a monitor (VG248, ASUS) 57 cm in front of the subject. An eye tracker (iView X Hi-Speed Primate, SMI) was used to monitor the monkey's eye position. A Matlab-based toolbox MonkeyLogic (NIMH) was used for experimental control.

During the recording session, the subject was first required to fixate at the center of the screen for 500 ms, and then an emotional face was presented for 800 ms. The subject could get a juice reward after successfully keeping fixation during the presenting period. In the meantime, the electrodes in the micro-drive (SC32, Gray Matter Research) were advanced carefully by tuning the bonded screws in steps of one-quarter to one-half turns (8 turns/mm). A 128-channel electrophysiological recording system (OmniPlex, Plexon Inc.) was used to monitor and record neural activities. Signals were filtered between 250 Hz and 5 kHz to identify spiking activity.

## Supplementary Material

nwae381_Supplemental_File
