# Supplementary material for: Genetic and neuronal basis for facial emotion perception in humans and macaques
Source: Natl Sci Rev. 2024 Nov 8;11(11):nwae381. doi: 10.1093/nsr/nwae381 (PMC11614104; doi:10.1093/nsr/nwae381)
Supplement: nwae381_Supplemental_File [file nwae381_supplemental_file.docx]

Supplementary Materials

**Genetic and Neuronal Basis for Facial Emotion Perception in Humans and Macaques**

Wang *et al.*

*Corresponding authors (Ji Dai: ji.dai@siat.ac.cn; Yi Jiang: yijiang@psych.ac.cn)

**MATERIALS AND METHODS**

**Twins-based behavioral experiment**

*Procedure for behavioral experiment*

The gender type of the face stimuli was counterbalanced across the seven conditions, and all test trials were presented in a new random order for each observer. It should be noted that the test trials can also be constructed to form seven new conditions: 20, 30, 40, 50, 60, 70, and 80% intensity of femininity.

*Behavioral data analysis*

The behavioral performance for Experiments 1 and 2 was measured by the difference limen (DL), which can reflect the emotion or gender discrimination sensitivity. A smaller DL value indicated greater sensitivity among participants in discerning emotion or gender. In Experiment 1, the DL was significantly different between the emotion condition and the gender condition (0.56 vs. 0.67, *t*_241_=-3.18, Cohen’s *d* = 0.25, *p* < 0.01). Moreover, the DL for the LSF condition was lower than that for the HSF condition in Experiment 2 (0.9 vs. 1.2, *t*_221_=-7.04, Cohen’s *d* = 0.52, *p* < 0.001), suggesting that participants were more sensitive to LSF content in facial emotion perception. This finding resonates well with previous studies showing the advantage of LSF processing in facial emotion perception [1-4].

For each observer under each test condition, we calculated the proportions of fearful or female responses to the morphed faces (Experiment 1), or fearful responses to the morphed HSF or LSF faces (Experiment 2). These results were then fit with a Boltzmann sigmoid function: F(x) = 1/(1+exp[(x-x_0_)/w]), and the statistical analyses were conducted on the different limen (DL), which was estimated by half the interquartile range of the fitted function. DL was used to measure the emotion or gender discrimination sensitivity.

*Genetic data analysis*

To assess the relative importance of genetic and environmental factors to the total phenotypic variations of emotion and gender discrimination ability, univariate genetic analyses, as described in Neale and Cardon [5], were conducted with the statistical package OpenMx (version 2.19.6) [6] in R (version 4.1.1)[7]. Linear structural equation models were separately fitted to variance-covariance matrices for MZ and DZ twins. These models assume that phenotypic variation arises from the factors of additive genetic (A), common environmental (C), and non-shared environmental (E) influences. The effect of additive genetic factors (A) is assumed to be the sum of multiple genes (polygene) whose effects are small and additive to form a quantitative phenotype. Common environment (C) is the effect that makes twins alike not from heredity but the environment shared by family members. Non-shared environment (E) is the effect of the individually unique environment that makes twins different, such as differential parental treatment, prenatal or postnatal traumas, and peer group. Non-shared environment (E) also includes measurement errors. The twin study design operates under the assumption that both MZ and DZ twins share their environment equally, while MZ twins share 100% and DZ twins typically share about 50% of their genetic makeup. Consequently, if genetic factors influence facial emotion perception, the similarity in emotion recognition abilities should be greater among MZ twin pairs compared to DZ twin pairs. In the twin model, potential confounding variables like cultural factors, which may affect emotion recognition abilities, are included in the environmental component and can be classified as either shared or non-shared environmental factors. Shared environmental factors, such as family upbringing and cultural practices, affect both twins similarly, while non-shared environmental factors, like unique personal experiences, affect only one twin. By comparing monozygotic (MZ) and dizygotic (DZ) twins, we can estimate the heritability of emotion recognition abilities, distinguishing genetic influences from environmental influences, including cultural factors. It should be noted that these modals rely on certain assumptions, such as the equality of environments shared by MZ and DZ, and that they do not account for gene-environment interactions.

To reveal which factors significantly contribute to phenotypic variance, different models were systematically compared in terms of goodness-of-fit statistics and parsimony. These are the ACE model in which phenotypic covariances are explained by A, C, and E; the AE model explained by just A and E; the CE model explained by just C and E; and the E-only model. Different models were compared using Akaike’s Information Criteria (AIC) which reflects a model’s goodness of fit as well as its parsimony, and the model that results in the smallest AIC is regarded as the best.

**Twins-based MRI Experiment**

*Image acquisition*

For each participant a high-resolution T1-weighted anatomical scan was acquired using a magnetization-prepared rapid acquisition gradient echo sequence with the following parameters: repetition time (TR) = 6.89 ms, echo time (TE) = 2.98 ms, flip angle = 8°, acquisition matrix = 256 * 256, and field of view (FOV) = 256 mm * 256 mm. We acquired 176 sagittal slices with a thickness of 1 mm. Functional T2* echoplanar images (EPI) were acquired using a sequence with the following parameters: TR = 2000 ms, TE = 30 ms, flip angle = 90°, acquisition matrix = 64 * 64, and FOV = 220 mm * 220 mm. We acquired 35 axial slices with a thickness of 3.5 mm and a gap of 0.5 mm. During the scan, which lasted 8 minutes, participants were instructed to keep their eyes closed and not focus their thoughts on anything.

*fMRI data preprocessing*

All pre-processing of the data was performed using the Data Processing Assistant for Resting-State fMRI (DPARSF 4.3, <http://rfmri.org/dpabi>) [8], which is based on Statistical Parametric Mapping (SPM12; http://www.fil.ion.ucl.ac.uk/spm, Wellcome Center for Human Neuroimaging, London, United Kingdom) and the Resting-State fMRI Data Analysis Toolkit (REST 1.8, <http://www.restfmri.net>) [9]. To make the longitudinal magnetization reach a steady state, we discarded the first 10 volumes. Then, corrections for slice timing and head motion were performed. Next, we implemented segmentation of the T1 map to generate the gray matter (GM), white matter (WM), cerebrospinal fluid (CSF) regions[10], nuisance variable regression, and spatial normalization with 2-mm cubic voxels. The nuisance variables included 24 motion parameters (6 head motion parameters, 6 head motion parameters one time point ahead, and 12 corresponding squared items), CSF and WM regions from the segmented individual [11], and linear and quadratic trends [12]. The volume-based framewise displacement (FD) was calculated to quantify head motion [13-15]. Two subjects were excluded from further analyses because their head motion exceeded 2.5mm or 2.5 degrees.

*Low-frequency fluctuations of the amygdala*

After preprocessing, the time course for each voxel was transformed into a frequency domain using the fast Fourier transform algorithm. For each voxel, the fractional amplitude of low-frequency fluctuations (fALFF) was calculated by dividing the sum of the amplitudes of the low-frequency part of the spectrum (0.01 - 0.1 Hz) by the sum of the amplitudes of the whole frequency spectrum. Demean was performed on fALFF.

*Genetic data analysis*

Univariate genetic analyses were conducted for amygdala volume and the fALFF of the amygdala. In order to further examine genetic and environmental contributions to the covariance between the emotion discrimination ability of LSF faces and the fALFF of the amygdala, bivariate genetic analysis was applied to the twin data using the Mx program. The bivariate correlated factors model was fit to the covariance matrices. This model allows the covariance of two phenotypes to be partitioned into covariance that is due to additive genetic, common environmental and non-shared environmental factors. Three parameters were calculated to respectively estimate the additive genetic (*r*_g_), common environmental (*r*_c_) and non-shared environmental (*r*_e_) correlations. The additive genetic correlation (*r*_g_) indicates the degree to which genetic influences on one phenotype overlap with those on the second phenotype (independent of their individual heritability). The sub-models, in which *r*_g_, *r*_c_, or *r*_e_ was dropped, were tested and compared with the full model to determine if the three parameters (*r*_g_, *r*_c_, and *r*_e_) were significant. A significant difference in chi-square indicates a worse fit and the parameter (*r*_g_, *r*_c_, or *r*_e_) dropped from the model is significant and should be retained in the model. Based on the univariate analyses of the emotion discrimination ability of LSF faces and the fALFF of the amygdala, the bivariate AE model was fitted.

**Monkey electrophysiological Experiment**

*Anesthesia and surgeries*

All surgical procedures were performed using sterile methods while the subject was anesthetized. For general anesthesia, monkeys were first administered with atropine (0.05 mg/kg, intramuscular) to decrease bronchial secretions, and then ketamine (15 mg/kg, intramuscular) and propofol (6 mg/kg, i.v.) were given successively to induce and maintain anesthesia. Electrocardiography, heart rate, oxygen saturation (SpO2), and rectal temperature were continuously monitored (Mindray, uMEC7).

The recording chamber (Form-fitting, PEEK) and the micro-drive (SC32-42mm, both from Gray Matter Research, Montana) were implanted in a two-step procedure following the product manual. In brief, firstly the recording chamber was implanted following the guide of T1-weighted MRI images (3T Tim Trio scanner, Siemens). Then the animal was run through a second MRI scan with a grid filled with Vitamin E to confirm the location of the chamber and register the position of the electrodes. Two weeks after the first surgery, a craniotomy was performed and the micro-drive was installed vertically above the amygdala [16].

*Data analysis and statistics*

Spikes were sorted offline using Offline Sorter (Version 4.4.2, Plexon Inc.) following the user manual to identify single units. K-means was used as the automatic sorting method. The peri-stimulus time histograms (PSTHs) of spike trains for each neuron across different conditions were obtained after aligning to the stimulus onset. The bin width of PSTH was set to 30 ms. Neurons with distinct visual responses to faces were then classified as LSF-preferred or HSF-preferred neurons according to the ratio of the average response intensity to low-SF faces vs. to high-SF faces. A ratio less than 1.0 indicated an HSF-preferred neuron. Otherwise, an LSF-preferred neuron. For each classified neuron, the responses were normalized using (*R* - *R_min_*)/(*R_max_* - *R_min_*), where *R* represented the original response, and *R_min_* and *R_max_* represented the minimum and maximum response across all conditions during the presenting period, respectively. Following linear interpolation, the normalized response curves with 1 ms resolution were used to extract the peak response. An average of 130 to 230 ms after stimulus onset was taken as the mean of peak response.

All statistical analyses were conducted using SPSS (version 25.0, IBM Corporation). The two-way analysis of variance (ANOVA) was used to compare neural responses among different conditions (spatial frequency × emotion) for the LSF-preferred and HSF-preferred groups. The paired *t*-test was used for further post-hoc tests.

**References**

1. Vuilleumier P, Armony JL, Driver J *et al.* Distinct spatial frequency sensitivities for processing faces and emotional expressions. *Nat Neurosci*. 2003; **6**(6): 624-631. doi: 10.1038/nn1057

2. Willenbockel V, Lepore F, Nguyen DK *et al.* Spatial frequency tuning during the conscious and non-conscious perception of emotional facial expressions–an intracranial ERP study. *Frontiers in psychology*. 2012; **3**: 237.

3. Vlamings PH, Goffaux V, Kemner C. Is the early modulation of brain activity by fearful facial expressions primarily mediated by coarse low spatial frequency information? *Journal of vision*. 2009; **9**(5): 12-12.

4. Guyader N, Chauvin A, Boucart M *et al.* Do low spatial frequencies explain the extremely fast saccades towards human faces? *Vision research*. 2017; **133**: 100-111.

5. Neale MC, Cardon LR. *Methodology for genetic studies of twins and families*. New York, NY, US: Kluwer Academic/Plenum Publishers, 1992.

6. Boker S, Neale M, Maes H *et al.* OpenMx: an open source extended structural equation modeling framework. *Psychometrika*. 2011; **76**: 306-317.

7. R Core Team R. R: A language and environment for statistical computing. 2013.

8. Yan CG, Wang XD, Zuo XN *et al.* DPABI: Data Processing & Analysis for (Resting-State) Brain Imaging. *Neuroinformatics*. 2016; **14**(3): 339-351. doi: 10.1007/s12021-016-9299-4

9. Song XW, Dong ZY, Long XY *et al.* REST: A Toolkit for Resting-State Functional Magnetic Resonance Imaging Data Processing. *Plos One*. 2011; **6**(9). doi: ARTN e2503110.1371/journal.pone.0025031

10. Ashburner J, Friston KJ. Unified segmentation. *Neuroimage*. 2005; **26**(3): 839-851. doi: 10.1016/j.neuroimage.2005.02.018

11. Behzadi Y, Restom K, Liau J *et al.* A component based noise correction method (CompCor) for BOLD and perfusion based fMRI. *Neuroimage*. 2007; **37**(1): 90-101. doi: 10.1016/j.neuroimage.2007.04.042

12. Yan CG, Craddock RC, Zuo XN *et al.* Standardizing the intrinsic brain: towards robust measurement of inter-individual variation in 1000 functional connectomes. *Neuroimage*. 2013; **80**: 246-262. doi: 10.1016/j.neuroimage.2013.04.081

13. Power JD, Barnes KA, Snyder AZ *et al.* Spurious but systematic correlations in functional connectivity MRI networks arise from subject motion. *Neuroimage*. 2012; **59**(3): 2142-2154. doi: 10.1016/j.neuroimage.2011.10.018

14. Satterthwaite TD, Wolf DH, Loughead J *et al.* Impact of in-scanner head motion on multiple measures of functional connectivity: relevance for studies of neurodevelopment in youth. *Neuroimage*. 2012; **60**(1): 623-632. doi: 10.1016/j.neuroimage.2011.12.063

15. Van Dijk KR, Sabuncu MR, Buckner RL. The influence of head motion on intrinsic functional connectivity MRI. *Neuroimage*. 2012; **59**(1): 431-438. doi: 10.1016/j.neuroimage.2011.07.044

16. Shan L, Yuan L, Zhang B *et al.* Neural Integration of Audiovisual Sensory Inputs in Macaque Amygdala and Adjacent Regions. *Neurosci Bull*. 2023. doi: 10.1007/s12264-023-01043-8

**Table S1.** Results of univariate genetic analyses for the full models and the best-fitting submodels of the behavior experiments.

|  | model | a^2^ (95% CI) | c^2^ (95%CI) | e^2^ (95%CI) | AIC |
| --- | --- | --- | --- | --- | --- |
| Emotion | ACE | 0.26(0.03-0.47) | 0(0-0.28) | 0.74(0.53-0.97) | 462.74 |
|  | AE | 0.26(0.03-0.47) |  | 0.74(0.53-0.97) | 460.74 |
| Gender | ACE | 0.12(0-0.45) | 0(0-0.19) | 0.88(0.55-1) | 708.96 |
|  | E |  |  | 1(1-1) | 705.40 |
| LSF | ACE | 0.30(0.05-0.51) | 0(0-0.29) | 0.70(0.49-0.95) | 628.68 |
|  | AE | 0.30(0.05-0.51) |  | 0.70(0.49-0.95) | 626.68 |
| HSF | ACE | 0(0-0.51) | 0.38(0-0.53) | 0.62(0.47-0.79) | 727.72 |
|  | CE |  | 0.38(0.21-0.53) | 0.62(0.47-0.79) | 725.72 |

**Table S2.** Results of univariate genetic analyses for the full models and the best-fitting submodels of the amygdala volumes and fALFF.

|  | model | a^2^ (95% CI) | c^2^ (95%CI) | e^2^ (95%CI) | AIC |
| --- | --- | --- | --- | --- | --- |
| Volumes | ACE | 0.59(0.13-0.85) | 0.18(0-0.59) | 0.23(0.14-0.38) | -129.22 |
|  | AE | 0.77(0.63-0.86) |  | 0.23(0.14-0.37) | -130.84 |
| fALFF | ACE | 0.42(0-0.62) | 0(0-0.49) | 0.58(0.38-0.84) | -929.46 |
|  | AE | 0.42(0.16-0.62) |  | 0.57(0.38-0.84) | -931.46 |


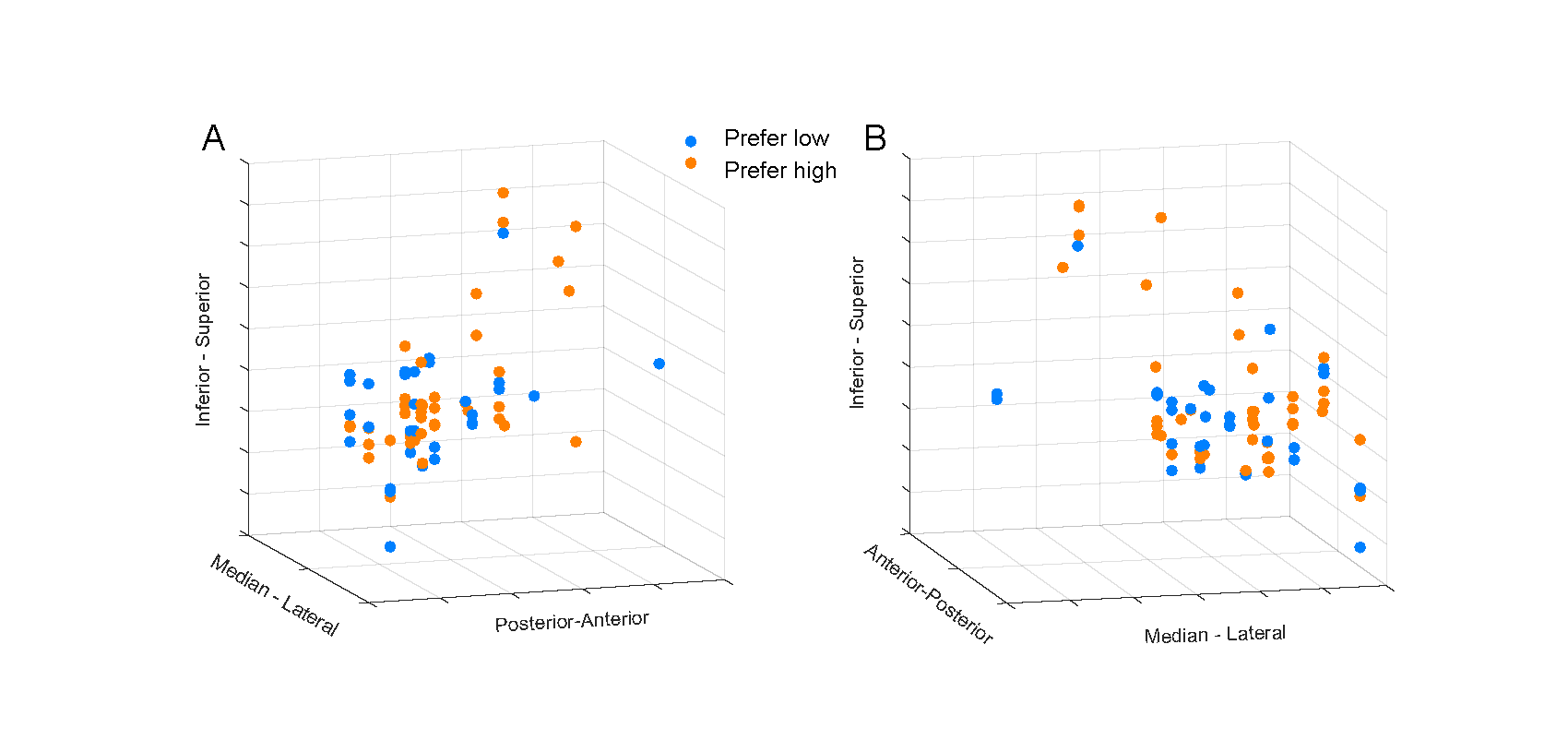


**Fig S1.** The distribution of the recording sites in the amygdala. (A) The posterior-anterior view. (B) The median-lateral view. Blue: LSF-preferred group; Yellow: HSF-preferred group.
